# The disintegrin echistatin in combination with doxorubicin targets high-metastatic human osteosarcoma overexpressing α_v_β_3_ integrin in chick embryo and nude mouse models

**DOI:** 10.18632/oncotarget.13497

**Published:** 2016-11-22

**Authors:** Yasunori Tome, Hiroaki Kimura, Naotoshi Sugimoto, Hiroyuki Tsuchiya, Fuminori Kanaya, Michael Bouvet, Robert M. Hoffman

**Affiliations:** ^1^ AntiCancer, Inc., San Diego, CA 92111, USA; ^2^ Department of Surgery, University of California, San Diego, CA 92103, USA; ^3^ Department of Orthopedic Surgery, Graduate School of Medicine, University of the Ryukyus, Okinawa, 903-0125 Japan; ^4^ Department of Orthopaedic Surgery, Graduate School of Medical Science, Kanazawa University, Kanazawa, 920-8641 Japan; ^5^ Department of Physiology, Graduate School of Medical Science, Kanazawa University, Kanazawa, 920-8641 Japan

**Keywords:** α, _v_β, _3_ integrin, echistatin, green fluorescent protein, red fluorescent protein, osteosarcoma, nude mice, orthotopic

## Abstract

Echistatin, a cyclic RGD peptide, which is an antagonist of α_v_β_3_ integrin (disintegrin), inhibited human osteosarcoma in the chick chorioallontoic membrane (CAM) model and tumor growth and pulmonary metastases in a nude mouse orthotopic model. A high-metastatic variant of human osteosarcoma, 143B-LM4, overexpressing α_v_β_3_ integrin was used. Tumor angiogenesis by high-metastatic variant 143B-LM4 cells in the CAM was significantly inhibited by echistatin (*P*<0.05) as was overall growth. A doxorubicin (DOX)-echistatin combination inhibited orthotopic tumor growth compared to untreated control (*P*<0.01) or DOX alone (*P*<0.05) in nude mice. Tumor-bearing mice treated with the DOX-echistatin combination survived longer than those treated with DOX alone or control PBS (*P*<0.01 and *P*<0.01, respectively). Echistatin also inhibited experimental lung metastasis of 143B-LM4 cells in nude mice. These results suggest that DOX in combination with a disintegrin has potential to treat osteosarcoma and that α_v_β_3_ integrin may be a target for osteosarcoma.

## INTRODUCTION

Osteosarcoma is a highly-metastatic cancer that is often fatal after failure of first-line chemotherapy [1]. Integrins are thought to play a major role in metastasis in this disease [2].

A high metastatic variant of 143B osteosarcoma cells (143B-LM4) was isolated in our laboratory by serial orthotopic passage and was found to overexpress α_v_β_3_ integrin, compared to parental cells [2].

**Figure 1:**
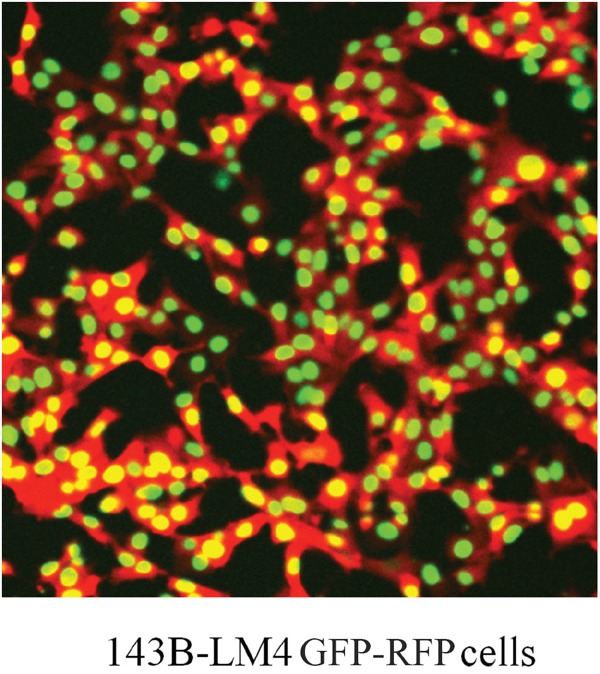
Dual-color 143B-LM4 human osteosarcoma cells expressing GFP in the nucleus and RFP in the cytoplasm *in vitro* The FV1000 confocal microscope (Olympus, Tokyo, Japan) was used to obtain images (20X).

We therefore decided to target α_v_β_3_ integrin as a potential improved therapy for osteosarcoma. Using the 143B-LM4 human osteosarcoma cell line, expressing RFP in the cytoplasm and GFP in the nucleus (143B-LM4-GEP-REP) (Figure 1), single cancer-cell seeding in the lung after i.v. injection in real time of osteosarcoma cells was imaged. Lung seeding by 143B-LM4 was greatly inhibited by the anti-β1 integrin monoclonal antibody, AIIB2. AIIB2 also significantly inhibited spontaneous lung metastasis and increased survival of mice with orthotopically-growing143B-RFP [2].

The *in vitro* efficacy of the disintegrin echistatin was tested on the 143B-LM4 high-metastatic variant, which over-expresses α_v_β_3_ integrin. Echistatin is an RGD cyclic peptide and an antagonist of α_v_β_3_ integrin. Echistatin inhibited cell proliferation, migration, invasion, and adhesion of 143B-LM4 cells. 143B-LM4 cell proliferation decreased after treatment with echistatin in a time-dependent and dose-dependent manner. *In vitro* migration and invasion as well as cell adhesion to vitonectin-coated dishes of 143B-LM4 cells were also inhibited by echistatin in a dose-dependent manner [3].

In the present study, we evaluated echistatin, targeting in combination with doxorubicin [4], against the 143B-LM4 cell line, in the chick embryo and in orthotopic and experimental-metastasis nude-mouse models.

## RESULTS AND DISCUSSION

### Efficacy of echistatin on the 143B-LM4 in the chick embryo

**Figure 2:**
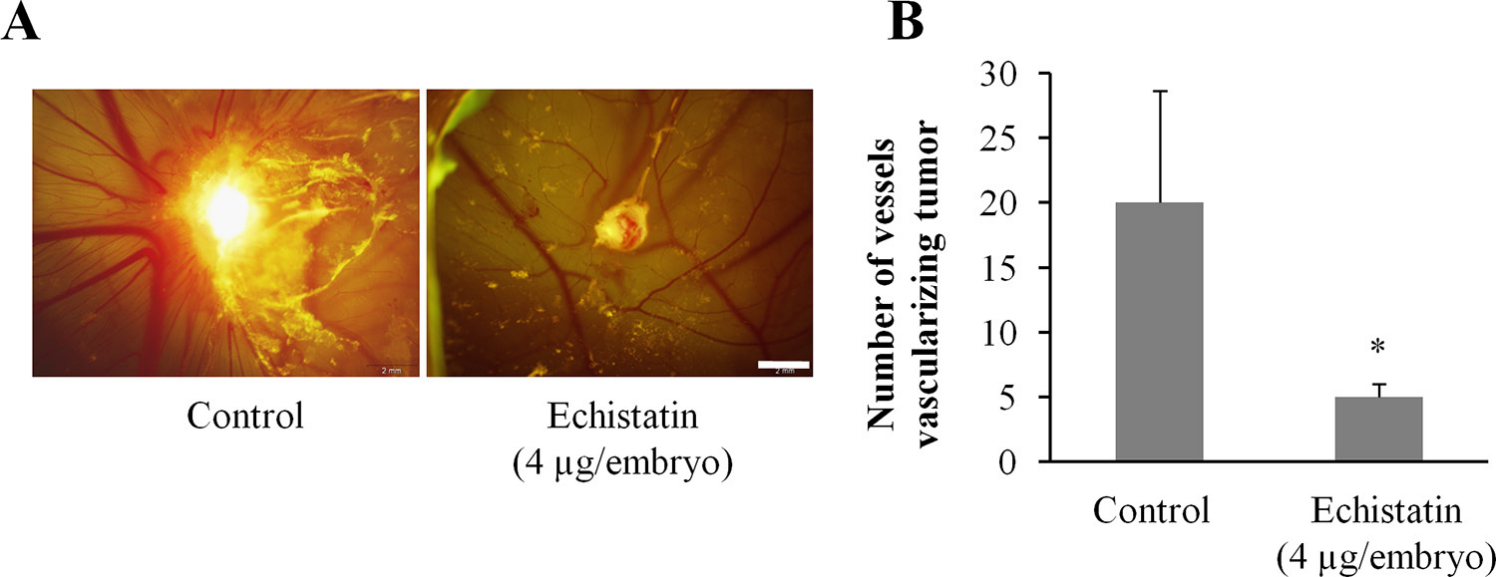
Efficacy of echistatin on tumor-induced angiogenesis and tumor growth in the chick chorioallantoic membrane (CAM) **A**. 143B-LM4-GEP-REP tumor fragments which grew on the CAM for 7 days were resected and implanted on another CAM which was treated with echistatin. Angiogenesis of the 143B-LM4 tumor was inhibited by echistatin compared with control (PBS). Tumor regression was also observed after treatment with echistatin. *Bar*: 2 mm. **B**. Quantitation of vessels vascularizing the tumor. Echistatin significantly decreased vascularization of the 143B tumor on the CAM (P<0.05). The OV100 variable-magnification fluorescence imager (Olympus, Tokyo, Japan) was used to obtain images. *Error bars* indicated SD.

Angiogenesis was initiated with fragments of the 143B-LM4 tumor implanted on the chorioallantoic membrane (CAM). After 24 hr, the embryos received a single inoculation of the echistatin or control PBS. Forty-eight hr after echistatin administration, tumor-induced angiogenesis by 143B-LM4 cells in the CAM was significantly inhibited by echistatin (P < 0.05) (Figure 2A, 2B). Tumor regression was also observed after treatment with echistatin compared to control PBS (Figure 2A, 2B).

### Efficacy of DOX-echistatin combination against 143B-LM4 in nude mice

**Figure 3:**
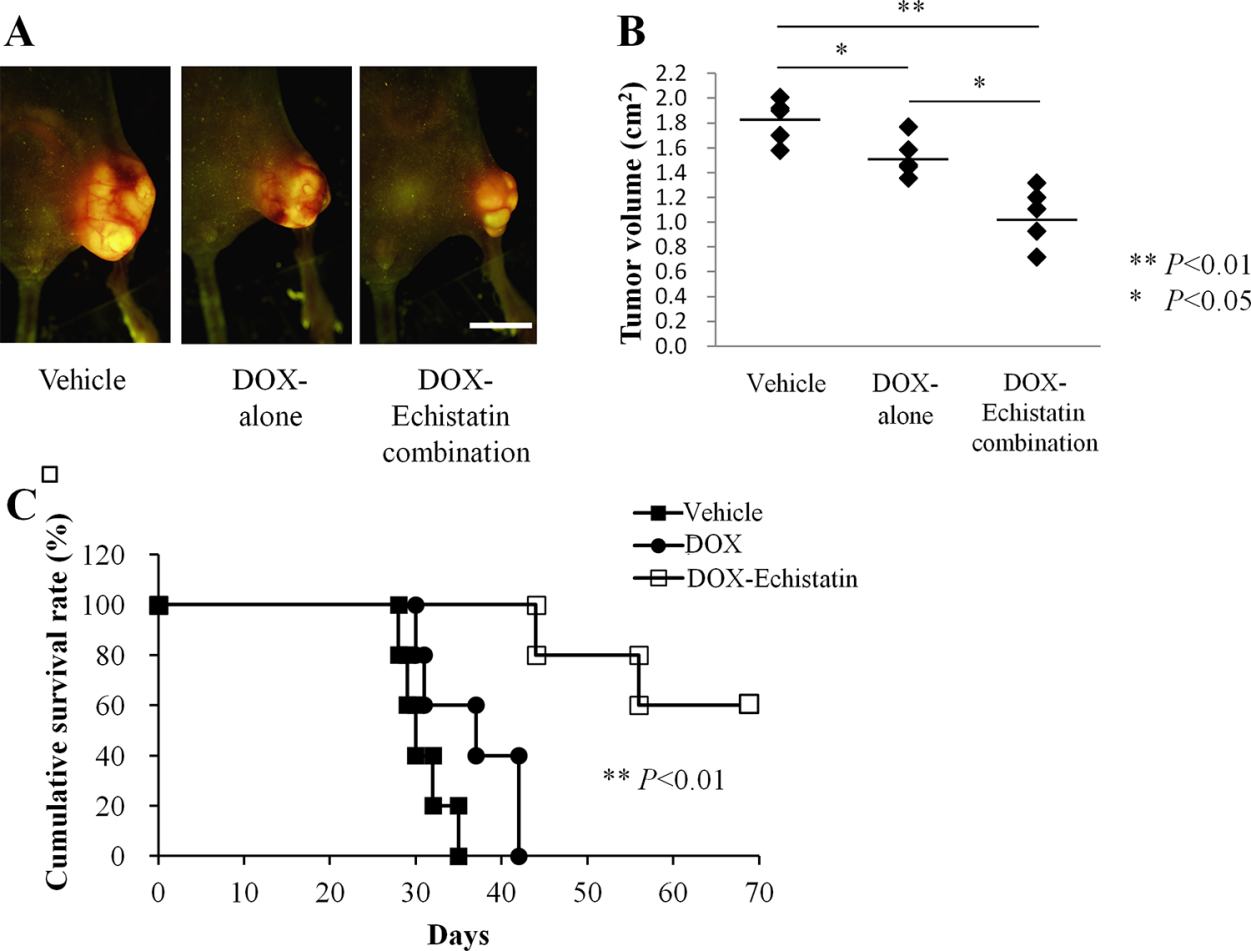
Efficacy of echistatin in combination with DOX on the 143B-LM4 orthotopic model in nude mice **A**. Efficacy of echistatin on metastasis in the 143B-LM4 orthotopic model in nude mice four weeks after transplantation in the tibia. The OV100 was used to obtain fluorescences images. *Bar*: 10 mm. **B**. Tumor volume four weeks after transplantation in the tibia. **C**. Kaplan-Meyer survival curves with the DOX-echistatin combination compared with control (PBS) or DOX-alone. *P*<0.01 for the DOX-echistatin combination on survival versus DOX- or PBS-treated animals.

The efficacy of a DOX-echistatin combination in the orthotopic 143B-LM4 tumor model in nude mice was determined. 143B-LM4 cells were transplanted in the tibia of 15 nude mice, and they were allowed to grow to palpable size. Mice were divided into three groups (5 mice PBS, 5 mice DOX, and 5 mice DOX-echistatin combination). Tumors were treated with intravenous injections of DOX (5 μg); DOX (5 μg) echistatin (4 μg); or PBS (100 μl) at one-week intervals. Four weeks after implantation of 143B-LM4 cells, the average primary tumor volume in the DOX-echistatin combination group was significantly smaller than that of the untreated control (*p* < 0.01) and that of the DOX-only (*p* <0.05) (Figure 3A & 3B). No adverse effects of DOX, DOX-echistatin combination, or PBS intravenous injection, such as body weight loss, were observed. Tumor-bearing mice treated with the DOX-echistatin combination survived longer than those treated with DOX alone or control PBS (P<0.01 and P<0.01, respectively) (Figure 3C). By day 35, all PBS control mice had to be euthanized. By 42 days, all mice treated with DOX were also euthanized due to tumor size. Two mice treated with the DOX-echistatin combination were euthanized by days 44 and 57, respectively. The remaining three mice treated with DOX-echistatin combination were alive up to three months from the time of tumor transplantation. No significant mouse body weight loss was observed in the treated groups compared to the control.

### Echistatin decreases 143B-LM4 osteosarcoma experimental lung metastases in nude mice

**Figure 4:**
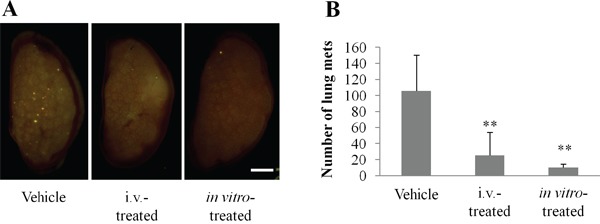
Echistatin decreases the number of experimental lung metastases in 143B-LM4 cells **A**. Fluorescence imaging of efficacy of echistatin on experimental metastasis of 143B-LM4-GFP-REP cells in nude mice one week after injection. The OV100 was used for fluorescence imaging. *Bar*: 2 mm. **B**. Quantitation of experimental lung metastases formed by 143B-LM4 cells. Echistatin decreased the number of experimental lung metastases in 143B-LM4 cells compared to untreated controls (*P*<0.01). *Error bars* indicate SD.

To determine whether echistatin could inhibit osteosarcoma experimental lung metastases, 143B-LM4 cells pretreated with echistatin were injected into the tail veil of nude mice. Untreated mice were used as the control. In another group on the following day, the mice were treated intravenously with echistatin (0.1 mg/kg). Lung samples were harvested from all of the mice one week after 143B-LM4 injection and imaged under fluorescence microscopy. The total number of metastases per lung was counted and averaged among the mice. Control mice had an average number of 105.8 pulmonary metastases. In contrast, mice treated with echistatin intravenously or *in vitro* had an average number of 25.3 or 10.2 experimental metastases, respectively (*P* < 0.01 and *P* < 0.01, respectively vs. untreated control) (Figure 4A & 4B).

Future studies will examine the cellular, histological and angiogenic effects of echistatin on both primary tumors and lung metastasis.

DOX is the one of the most frequently used drugs to treat osteosarcoma [5]. In the present study, there was a significant decrease in tumor size of a high-metastatic variant osteosarcoma, 143B-LM4 that overexpresses α_v_β_3_ integrin in mice treated with the combination of DOX and echistatin compared to DOX alone or untreated controls. These results suggest that DOX in combination with echistatin has clinical potential in osteosarcoma. Since echistatin is a disintegrin, the results of the present study suggest α_v_β_3_ integrin may be a target in osteosarcoma.

Previously-developed concepts and strategies of highly-selective tumor targeting can take advantage of molecular targeting of tumors, including tissue-selective therapy which focuses on unique differences between normal and tumor tissues [6–11].

## MATERIALS AND METHODS

### Cell cultures and chemicals

143B-LM4 cells expressing GEP in the nucleus and REP in the cytoplasm (143B-LM4-GEP-REP) [12] (Figure 1) were maintained with RPMI 1640 median (Irvine Scientific, Santa Ana, CA) containing 15% fetal bovine serum (FBS) (Omega Scientific, San Diego, CA) and 1% penicillin/streptomycin at 37°C in 5% CO_2_. Echistatin was purchased from Tocris Bioscience (Ellisville, MO), diluted with distilled water, and stored at -20°C.

### Angiogenesis on the chick chorioallantoic membrane

The chorioallantoic membrane (CAM) assay for tumor-induced angiogenesis was performed as previously described [13, 14]. Ten-day-old chick embryos were purchased from Mclntyre Poultry (Lakeside, CA) and were incubated at 37°C with 60% humidity. A small hole was made through the shell at the end of the egg directly over the air sac with the use of a small craft drill. A second hole was drilled on the broad side of the egg directly over embryonic blood vessels, as determined previously by candling. Negative pressure was applied to the original hole, which resulted in the CAM pulling away from the shell membrane and creating a false air sac. A 1.0 cm × 1.0 cm square window was cut through the shell over the dropped CAM with the use of a small model grinding wheel to create a window that allowed direct access to the underlying CAM.

143B-LM4-GEP-REP cells were seeded on the CAMs of 10-day-old chick embryos. A cell suspension of 143B-LM4-GFP-RFP (1×10^8^) was applied to the CAMs in a total volume of 30 μl RPMI 1640 medium with 15% FBS. The windows were sealed with tape, and the embryos were incubated for 7 days to allow growth of the human tumors. At the end of 7 days, the tumors were excised and trimmed free of surrounding CAM tissue. The tumors were then sliced into fragments of approximately 50 mg for use in the following angiogenesis assays.

Angiogenesis was induced in the chick embryo by implanting 50 mg fragments of 143B-LM4 tumor on the CAMs of 10-day-old embryos. After 24 h, echistatin (4 μg/embryo) or control PBS were injected. At the end of the-3-day incubation period, images were obtained with the OV100 Small Animal Imaging System (Olympus, Tokyo, Japan) and then angiogenesis was quantitated.

### Mice

Athymic nu/nu nude mice (AntiCancer Inc., San Diego, CA), 4–6 weeks old, were used in this study. All mouse surgical procedures and imaging were performed with the animals anesthetized by subcutaneous injection of a ketamine mixture (0.02 ml solution of 20 mg/kg ketamine, 15.2 mg/kg xylazine, and 0.48 mg/kg acepromazine maleate). The response of animals during surgery was monitored to ensure adequate depth of anesthesia. The animals were observed on a daily basis and humanely sacrificed by CO_2_ inhalation if they met the following humane endpoint criteria: severe tumor burden (more than 20 mm in diameter), prostration, significant body weight loss, difficulty breathing, rotational motion and body temperature drop. Animals were housed in a barrier facility on a high efficacy particulate arrestance (HEPA)-filtered rack under standard conditions of 12-hour light/dark cycles. The animals were fed an autoclaved laboratory rodent diet. All animal studies were conducted in accordance with the principles and procedures outlined in the National Institutes of Health Guide for the Care and Use of Animals under Assurance Number A3873-1.

### Nude mouse orthotopic metastatic model

143B-LM4 cells (5×10^5^) were transplanted into the tibia of the nude mice under the ketamine anesthesia, described above, as previously described [15]. One week after intra-tibial implantation, animals were separated into three groups and treated with doxorubicin (DOX) (0.25 mg/kg), a DOX (0.25 mg/kg)-echistatin (0.1 mg/kg) combination, or control PBS in each group five times at 1-week intervals. Tumor size was measured every other day and the mean tumor volume was calculated by the formula (square root of width×length/0.52) [16]. Mice were euthanized when tumor volume became approximately 2.0 cm^3^.

### The effect of echistatin on experimental lung metastases

To determine whether echistatin could inhibit experimental osteosarcoma pulmonary metastases, a total of 200 μl medium containing 143B-LM4 cells (1.5×10^6^) were injected into the tail vein of nude mice. The following day, animals were separated into two groups and treated with echistatin (0.1 mg/kg) or control PBS intravenously. In another experiment, 143B-LM4 cells treated with echistatin (1 μg/ml) prior to injection for 1 hr *in vitro* and then the cells were injected. One week after tumor injection, the lungs were harvested and imaged under fluorescence microscopy (OV100). The total number of metastases per lung was counted and averaged among the mice.

### *In vivo* imaging

The OV100 small animal imaging system (Olympus Corp., Tokyo, Japan), was used. The OV100 contains an MT-20 light source (Olympus Biosystems, Planegg, Germany) and DP70 CCD camera (Olympus) for subcellular imaging in live mice. The optics of the OV100 have been specially developed for macroimaging as well as microimaging with high light-gathering capacity. The instrument incorporates a unique combination of high numerical aperture and long working distance. Four individually optimized objective lenses, parcentered and parfocal, provide a 105-fold magnification range for seamless imaging of the entire body down to the subcellular level without disturbing the animal [17, 18].

Imaging was also performed with an FV 1000 laser-scanning confocal microscope (Olympus, Tokyo, Japan) with a XLUMPLFL 20× (0.95 NA) water-immersion objective. GFP was excited at 488 nm. Images were produced with FV10-ASW Fluoview software (Olympus) and ImageJ and were not modified beyond the standard adjustment of intensity levels [19].

### Statistical analysis

The data are presented as mean ± SD or mean ± SEM. Statistical analysis methods were the standard two-tailed Student's *t*-test for two data sets and ANOVA for multiple data sets. The animal survival data were analyzed using Kaplan-Meier survival analysis and log-rank test. *P* values of less than 0.05 were considered significant.
